# Supplementary figures and images for: Cannabinoid signaling modulation through JZL184 restores key phenotypes of a mouse model for Williams–Beuren syndrome
Source: eLife. 2022 Oct 11;11:e72560. doi: 10.7554/eLife.72560 (PMC9553213; doi:10.7554/eLife.72560)

# Figure 1

a

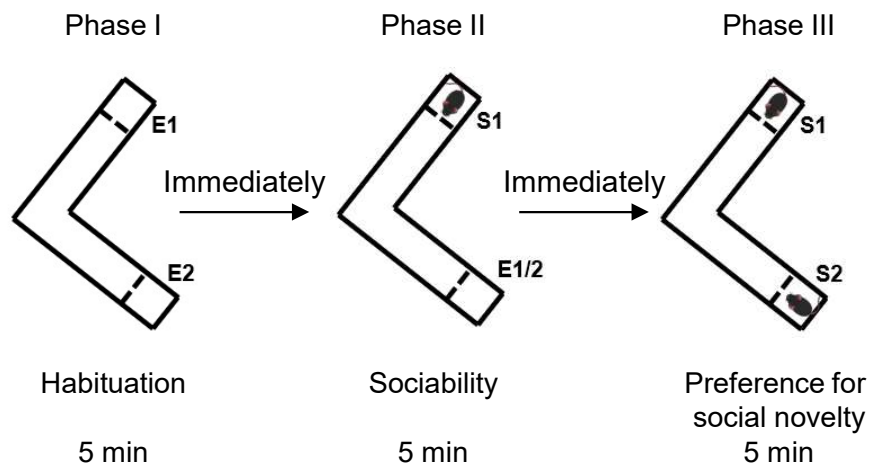

b

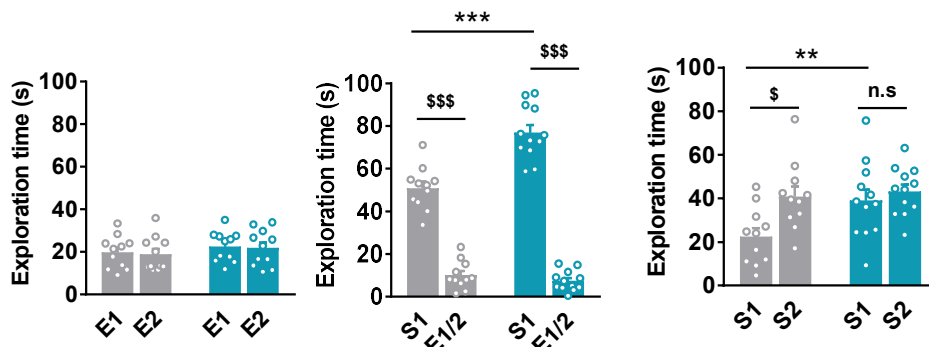

c

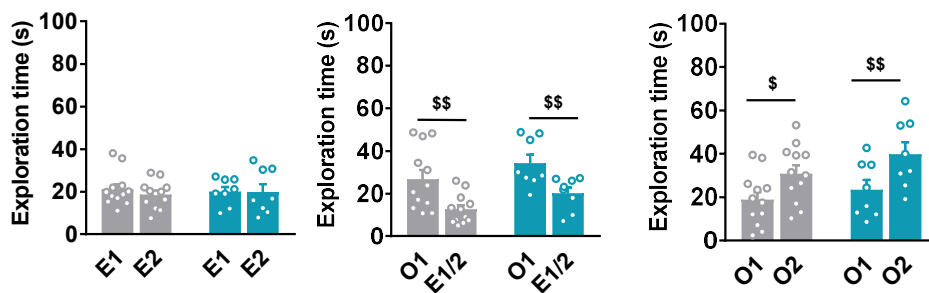

d

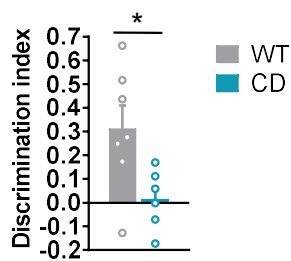

Supplement: Figure 1—source data 1. [file elife-72560-fig1-data1.zip › Figure 1-source data 1/Figure 1.pdf]

Figure 1-figure supplement 2

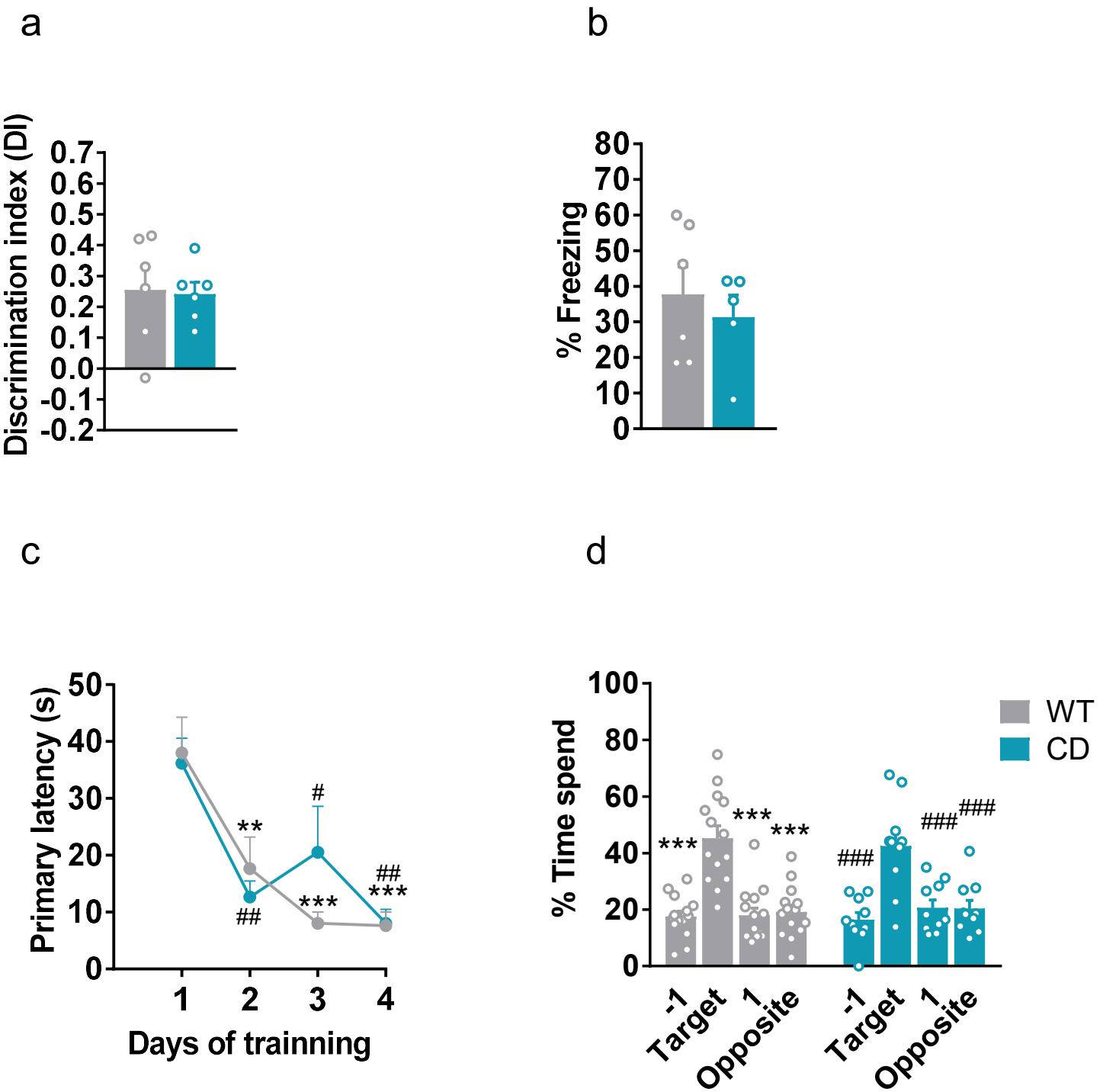

Supplement: Figure 1—figure supplement 2—source data 1. [file elife-72560-fig1-figsupp2-data1.zip › Figure 1-figure supplement 2-source data 1/Figure 1-figure supplement 2.pdf]

**Figure 2**

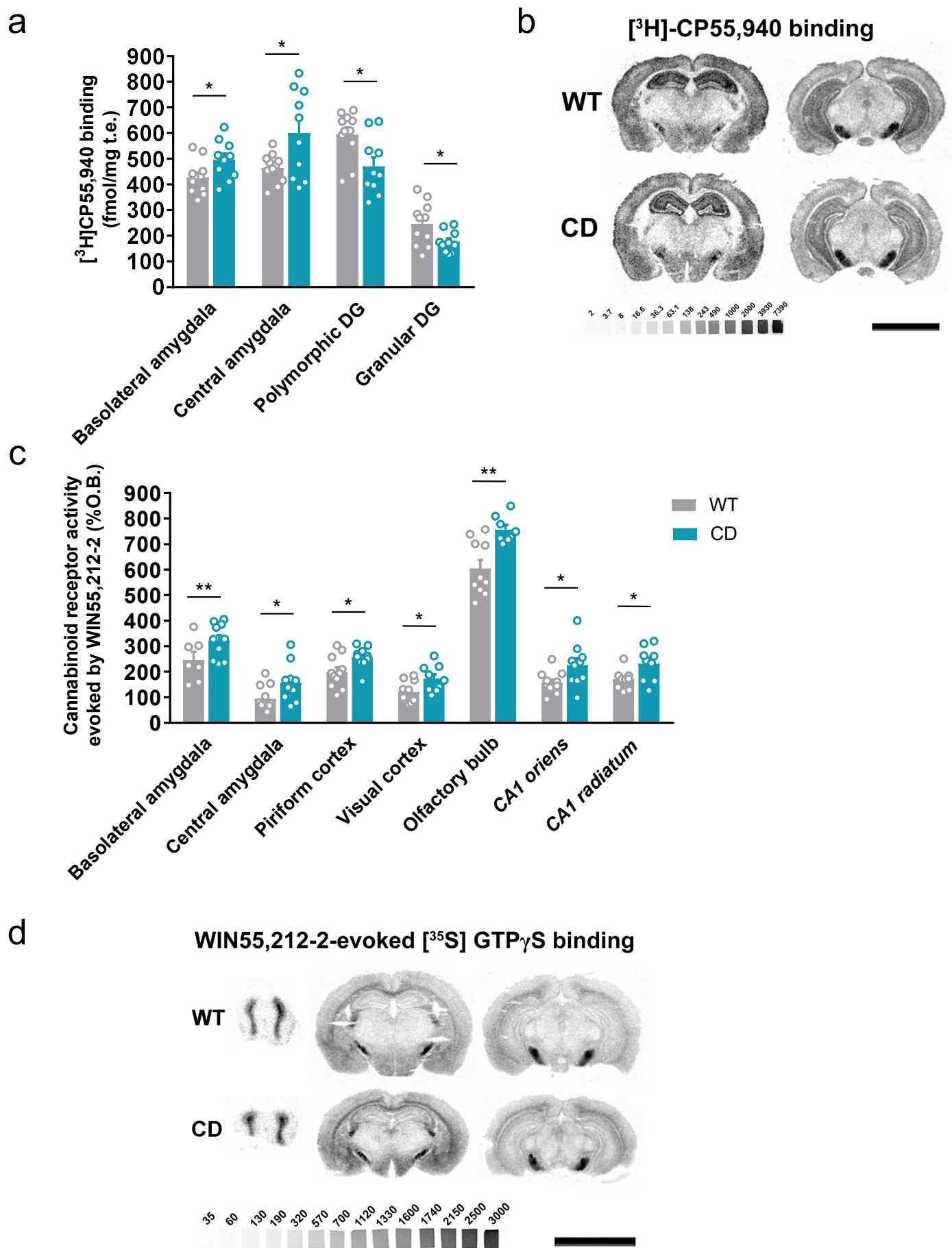

Supplement: Figure 2—source data 1. [file elife-72560-fig2-data1.zip › Figure 2-source data 1/Figure 2.pdf]

Figure 3

a

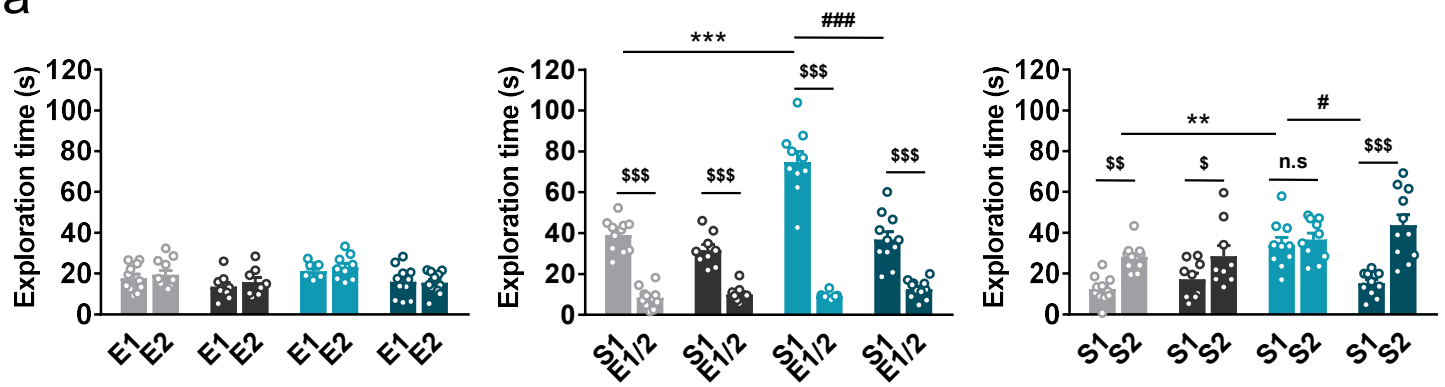

b

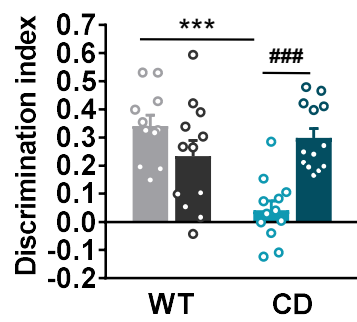

c

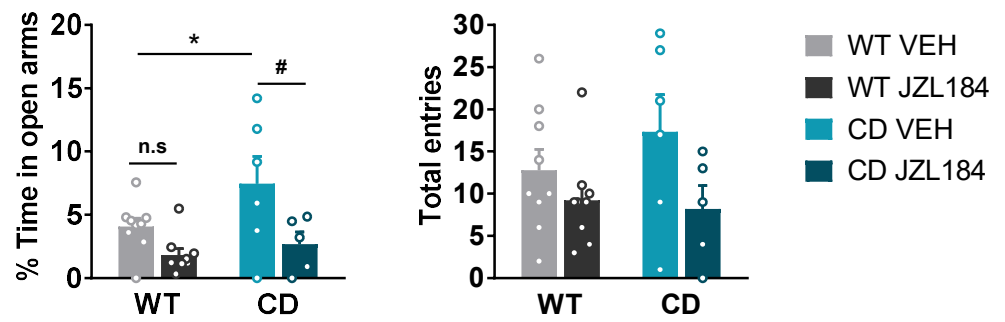

Supplement: Figure 3—source data 1. [file elife-72560-fig3-data1.zip › Figure 3-source data 1/Figure 3.pdf]

Figure 3-figure supplement 1

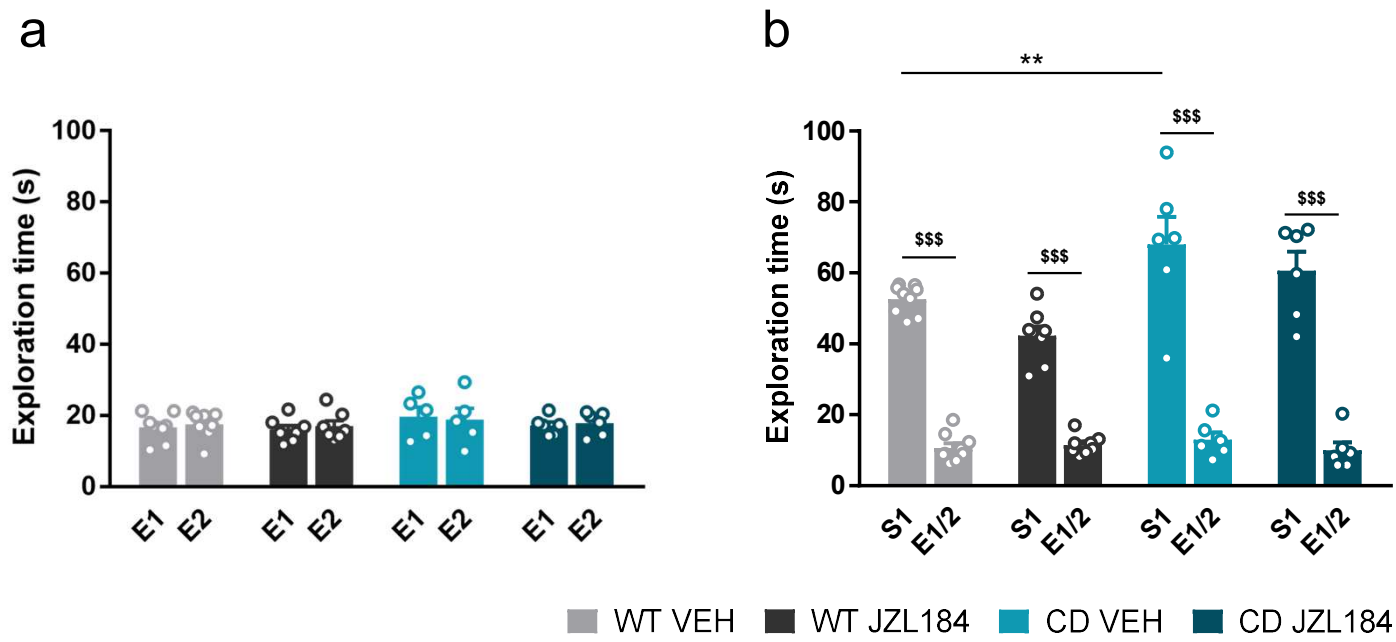

Supplement: Figure 3—figure supplement 1—source data 1. [file elife-72560-fig3-figsupp1-data1.zip › Figure 3-figure supplement 1-source data 1/Figure 3-figure supplement 1.pdf]

Figure 3-figure supplement 2

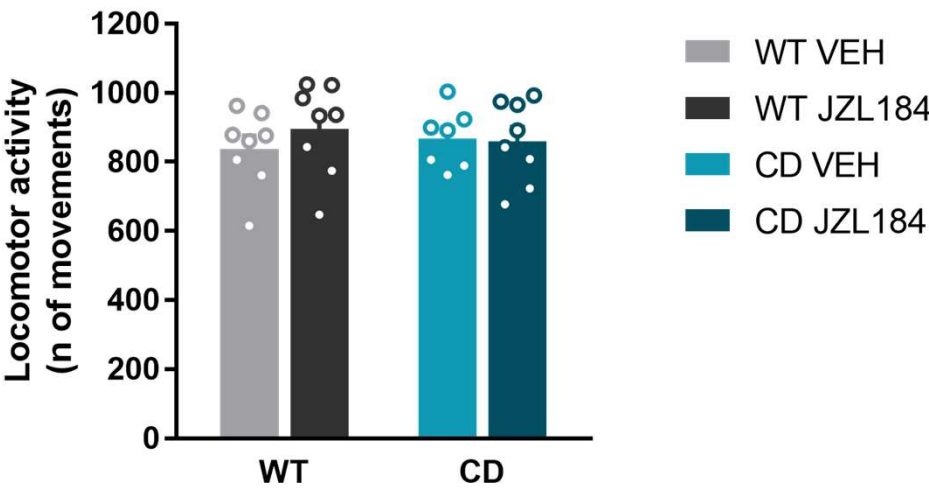

Supplement: Figure 3—figure supplement 2—source data 1. [file elife-72560-fig3-figsupp2-data1.zip › Figure 3-figure supplement 2-source data 1/Figure 3-figure supplement 2.pdf]

**Figure 4**

**a**

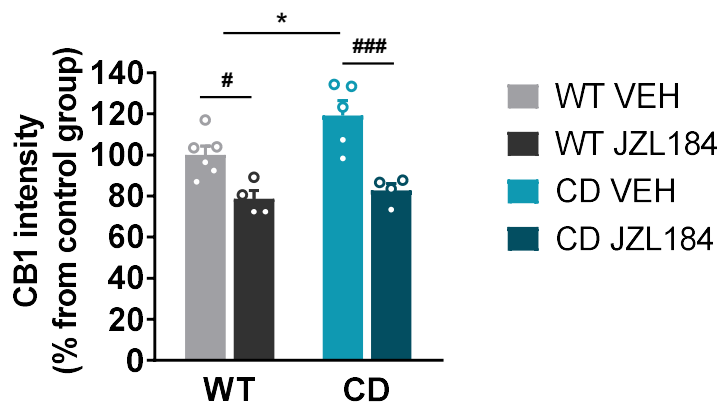

**b**

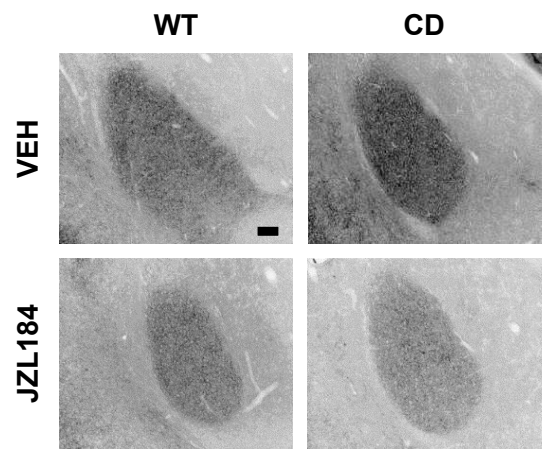

**c**

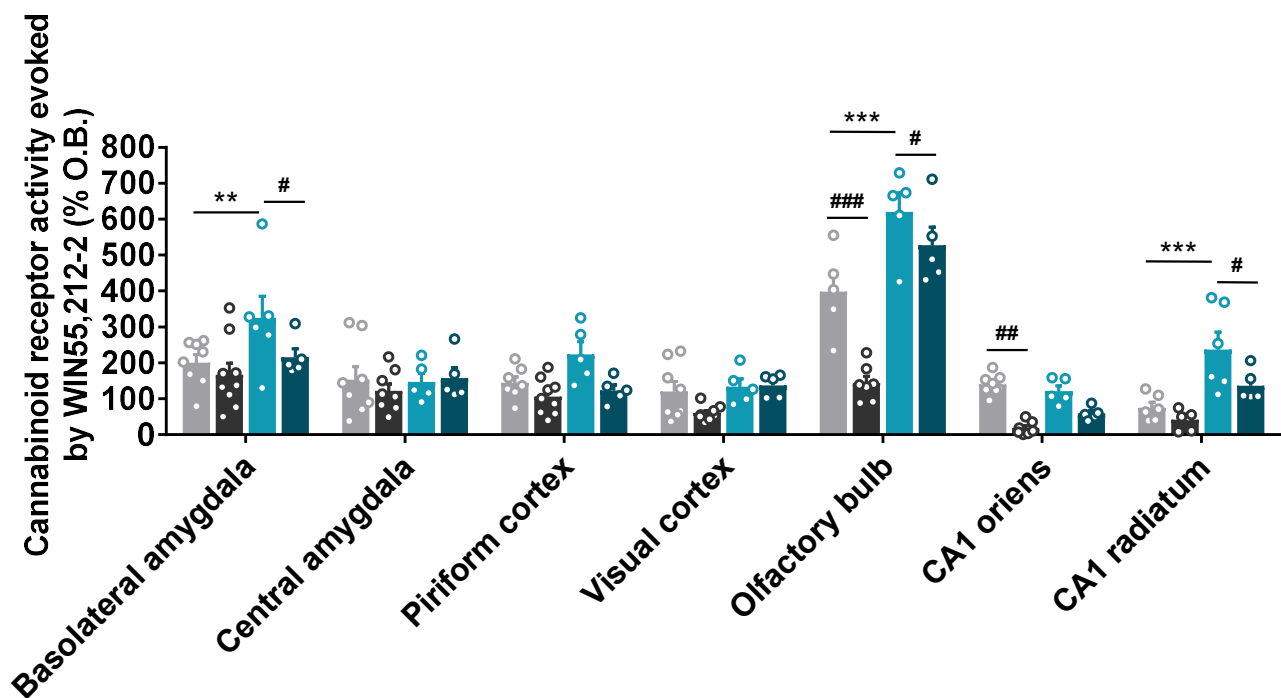

**d**

WIN55,212-2-evoked [<sup>35</sup>S] GTP<sub>γ</sub>S binding

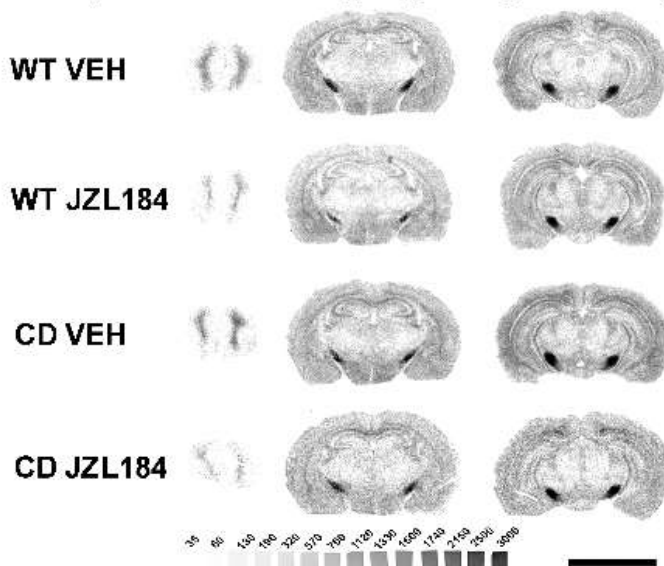

Supplement: Figure 4—source data 1. [file elife-72560-fig4-data1.zip › Figure 4-source data 1/Figure 4.pdf]

Figure 4-figure supplement 1

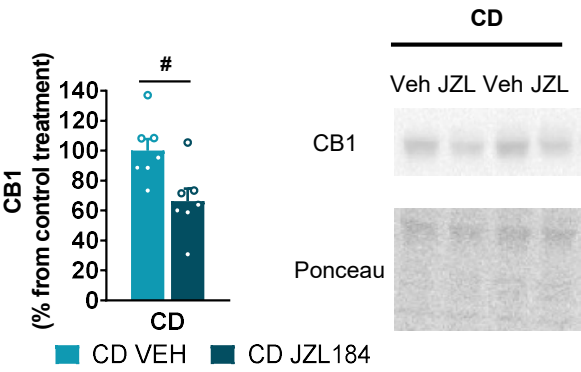

Supplement: Figure 4—figure supplement 1—source data 1. [file elife-72560-fig4-figsupp1-data1.zip › Figure 4-figure supplement 1-source data 1-source data 2-original files/Figure 4-figure supplement 1.pdf]

Figure 4-figure supplement 2

a

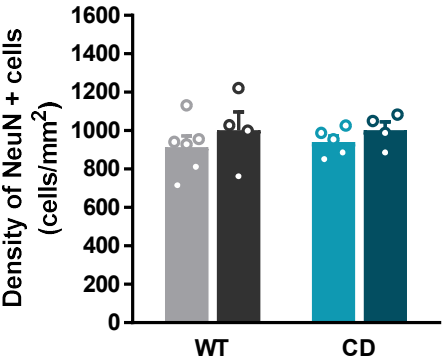

b

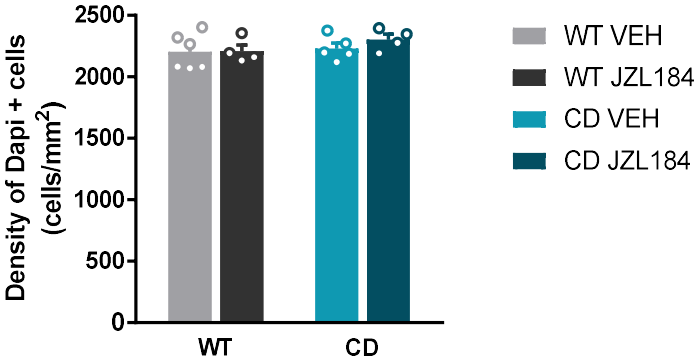

Supplement: Figure 4—figure supplement 2—source data 1. [file elife-72560-fig4-figsupp2-data1.zip › Figure 4-figure supplement 2-source data 1/Figure 4-figure supplement 2.pdf]

Figure 5

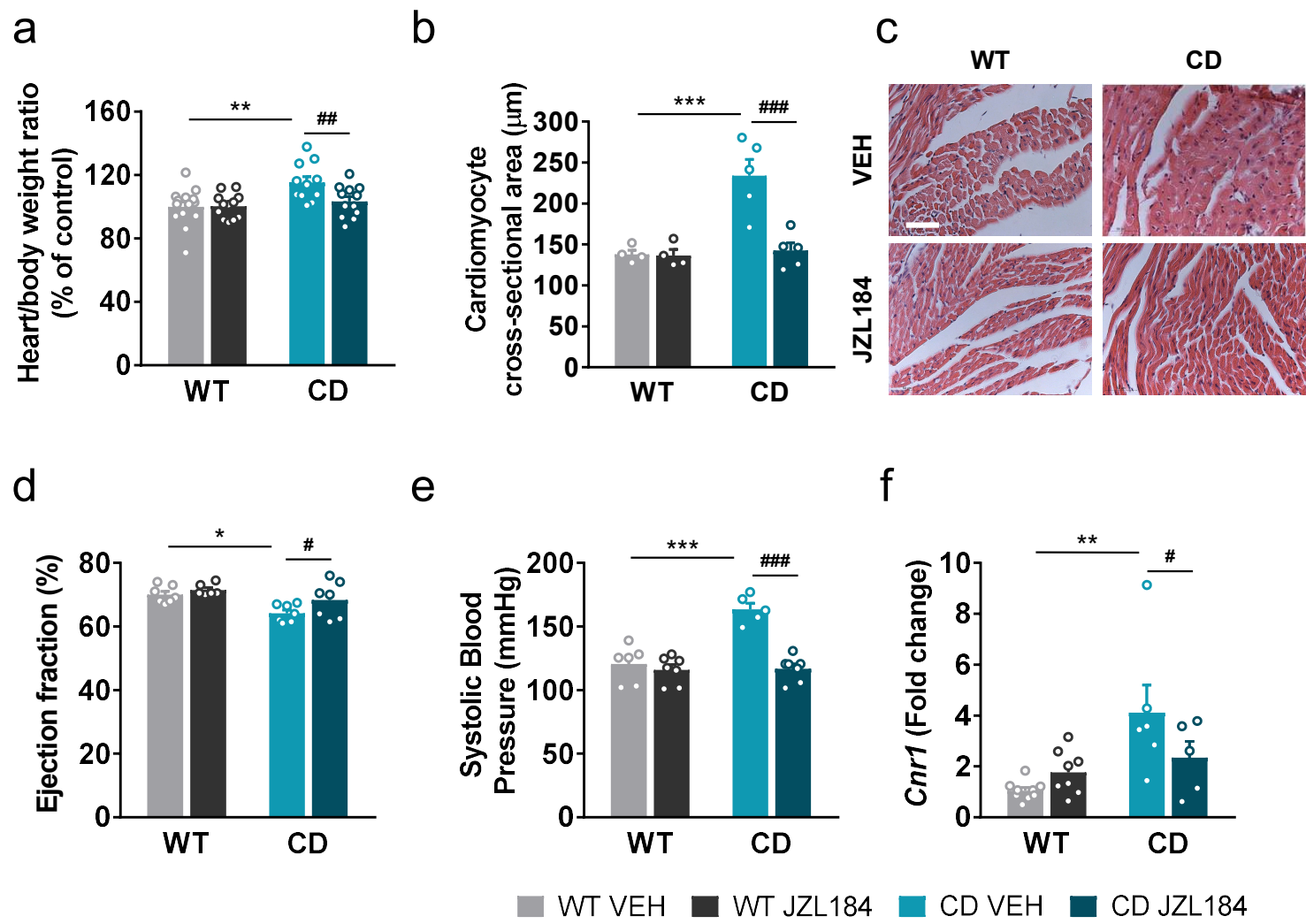

Supplement: Figure 5—source data 1. [file elife-72560-fig5-data1.zip › Figure 5-source data 1/Figure 5.pdf]

Figure 5-figure supplement 1

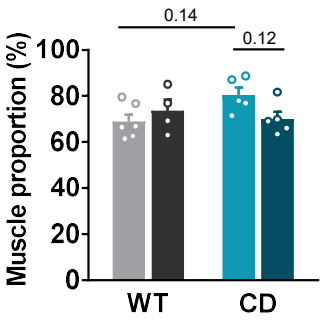

WT VEH  
WT JZL184  
CD VEH  
CD JZL184

VEH

JZL184

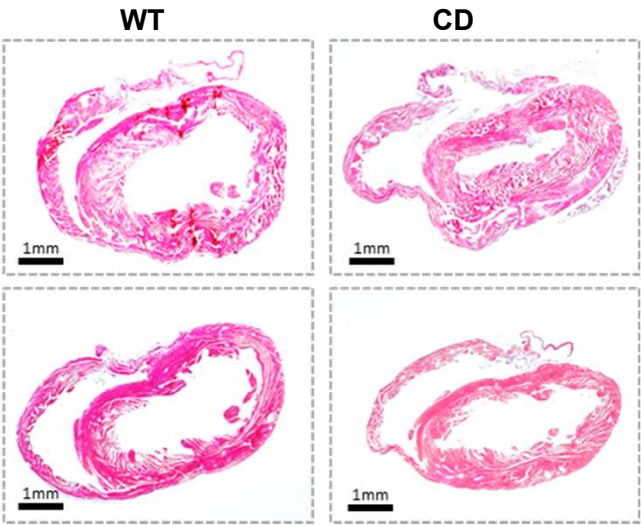

Supplement: Figure 5—figure supplement 1—source data 1. [file elife-72560-fig5-figsupp1-data1.zip › Figure 5-figure supplement 1-source data 1/Figure 5-figure supplement 1.pdf]

Figure 5-figure supplement 2

a

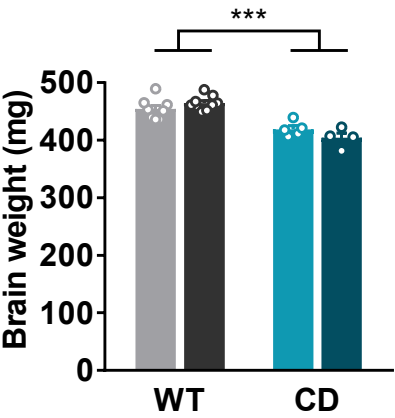

b

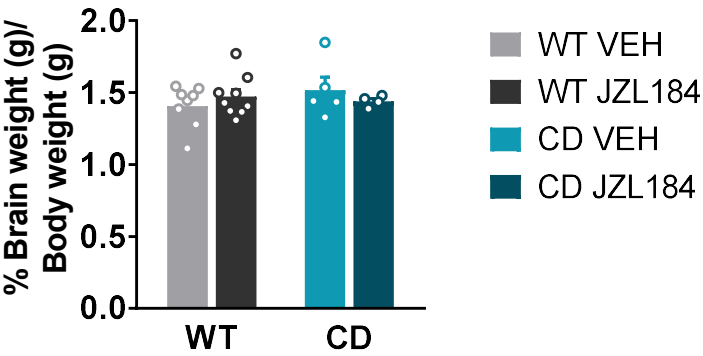

Supplement: Figure 5—figure supplement 2—source data 1. [file elife-72560-fig5-figsupp2-data1.zip › Figure 5-figure supplement 2-source data 1/Figure 5-figure supplement 2.pdf]

Figure 6

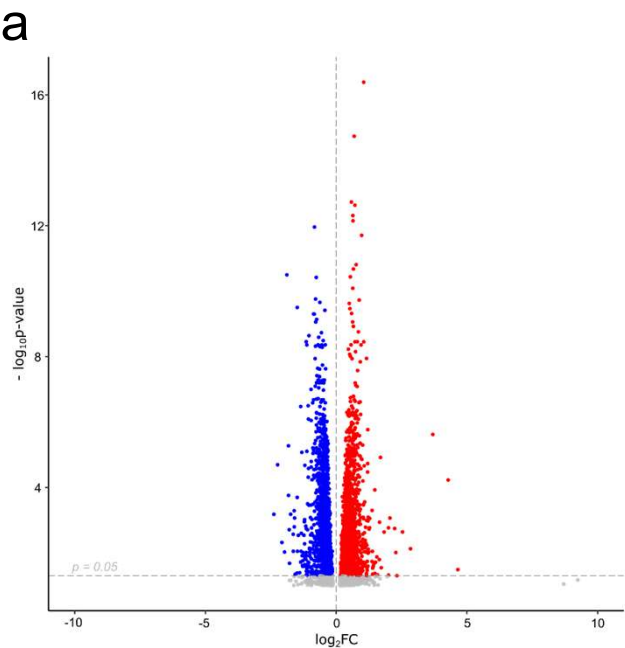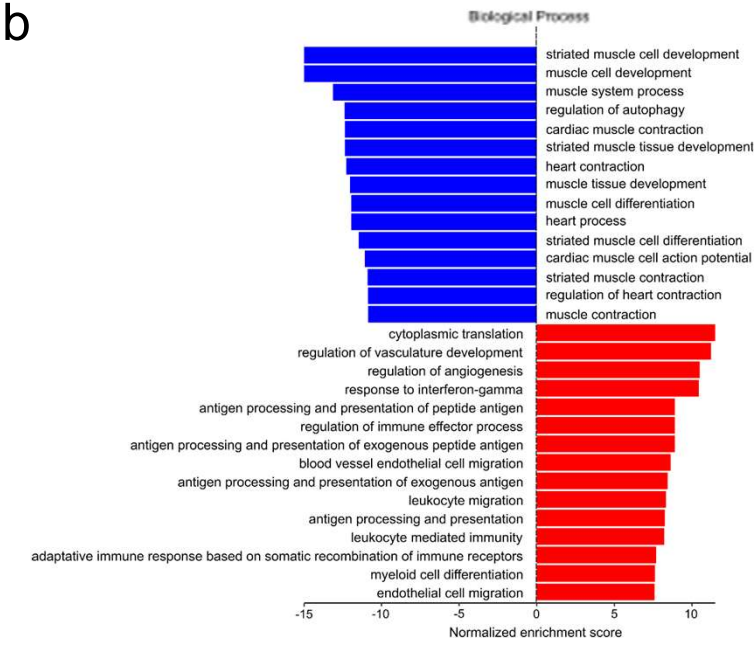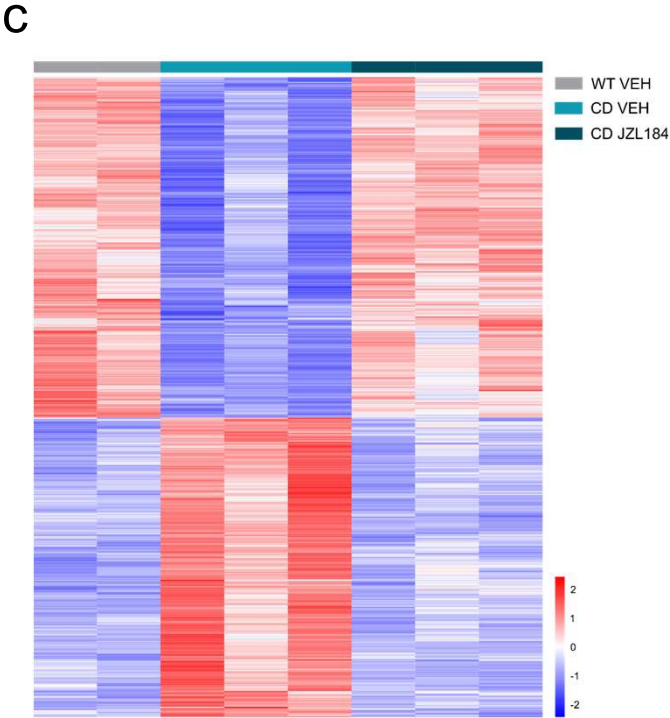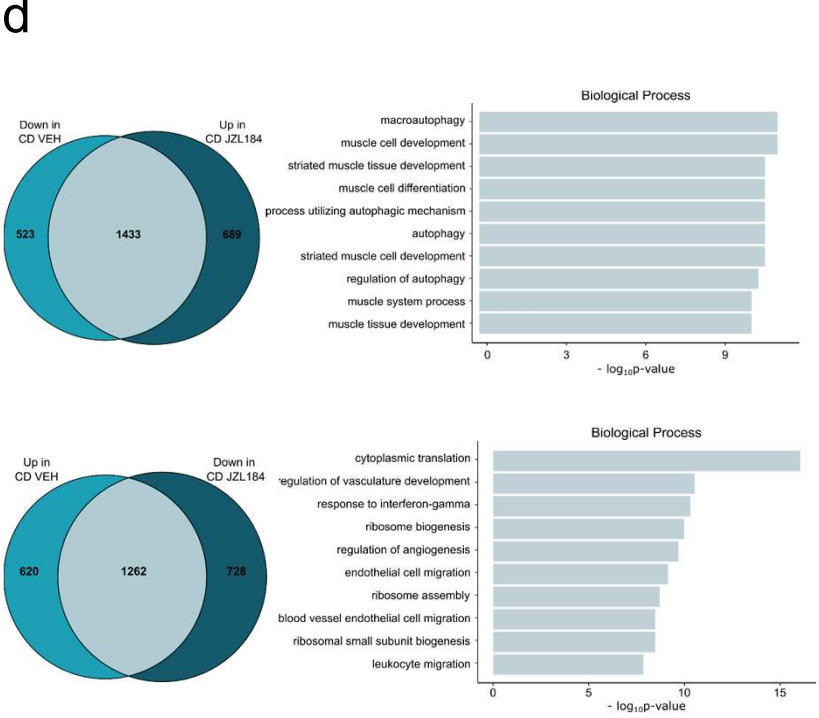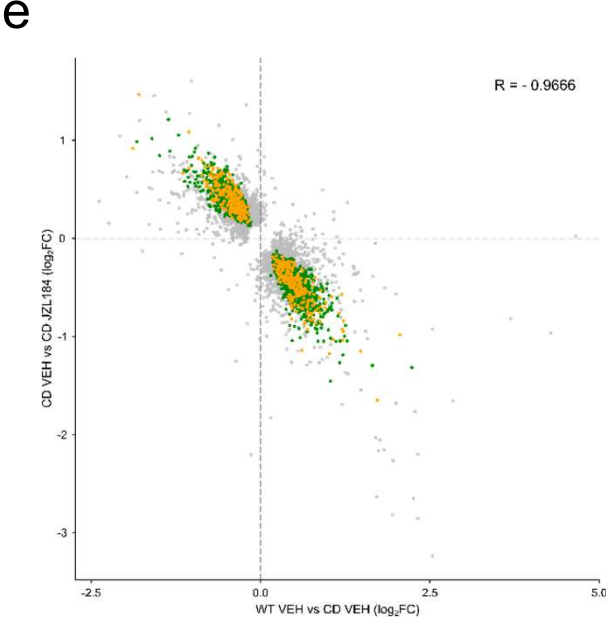

Supplement: Figure 6—source data 1. [file elife-72560-fig6-data1.zip › Figure 6-source data 1/Figure 6.pdf]

**Figure 6-figure supplement 1**

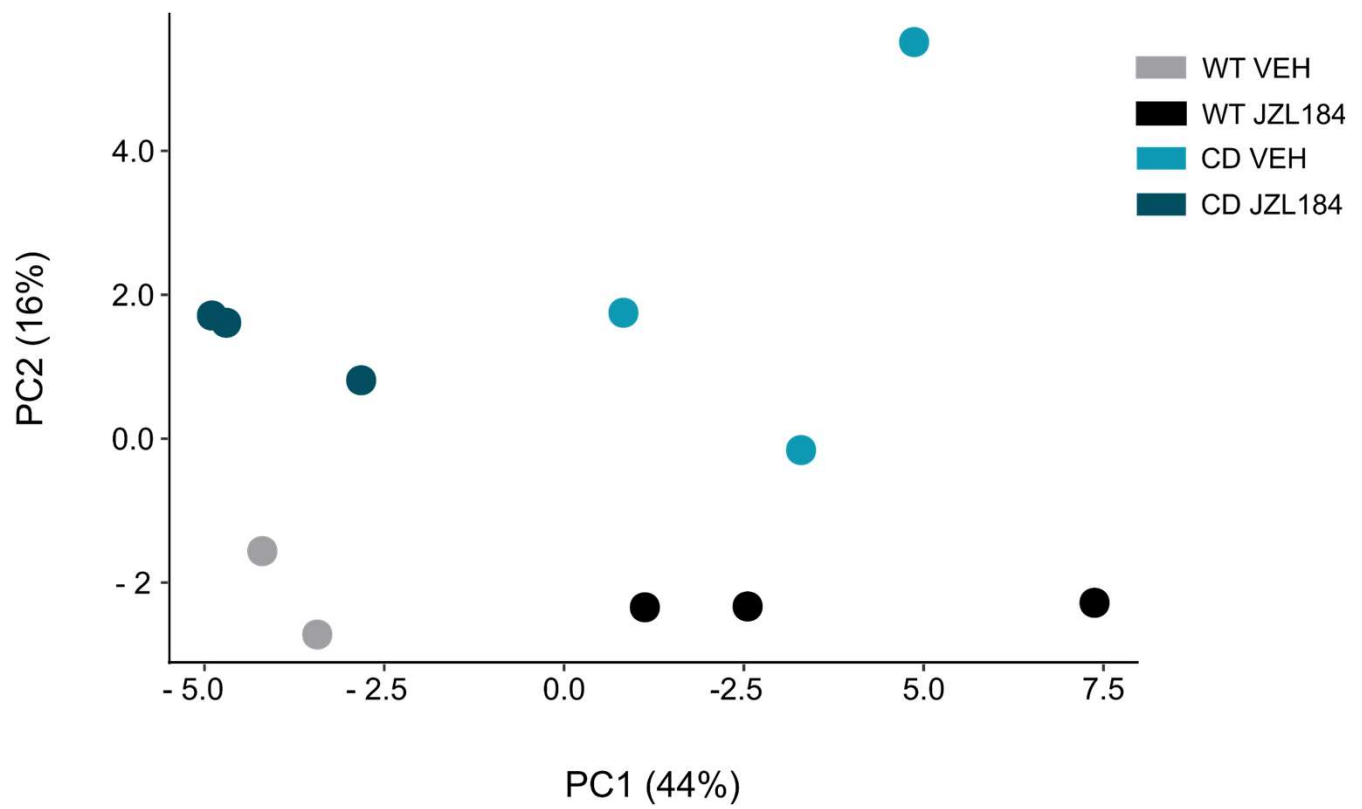

Supplement: Figure 6—figure supplement 1—source data 1. [file elife-72560-fig6-figsupp1-data1.zip › Figure 6-figure supplement 1-source data 1/Figure 6-figure supplement 1.pdf]
