# Supplementary material for: Cannabinoid signaling modulation through JZL184 restores key phenotypes of a mouse model for Williams–Beuren syndrome
Source: eLife. 2022 Oct 11;11:e72560. doi: 10.7554/eLife.72560 (PMC9553213; doi:10.7554/eLife.72560)

Figure 4-figure supplement 1- source data 2

CB1R

Name of the file: “WBS_AMG_VEH VS JZL_CB1 v2”

Gel A

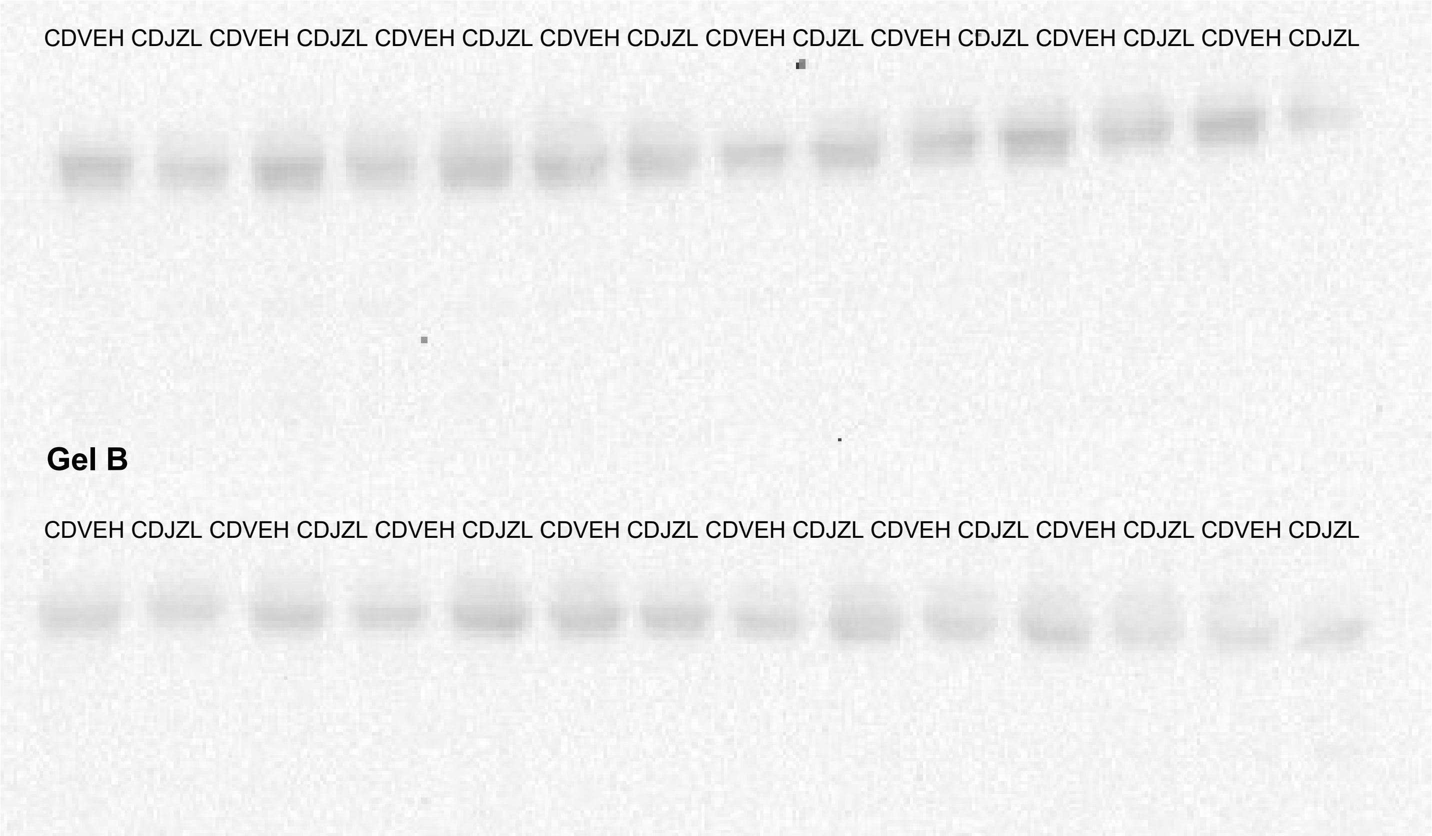

50 kDA

50 kDA

Ponceau

Name of the file: “ponceubaix2”

CDVEH CDJZL CDVEH CDJZL CDVEH CDJZL CDVEH CDJZL CDVEH CDJZL CDVEH CDJZL CDVEH CDJZL CDVEH CDJZL

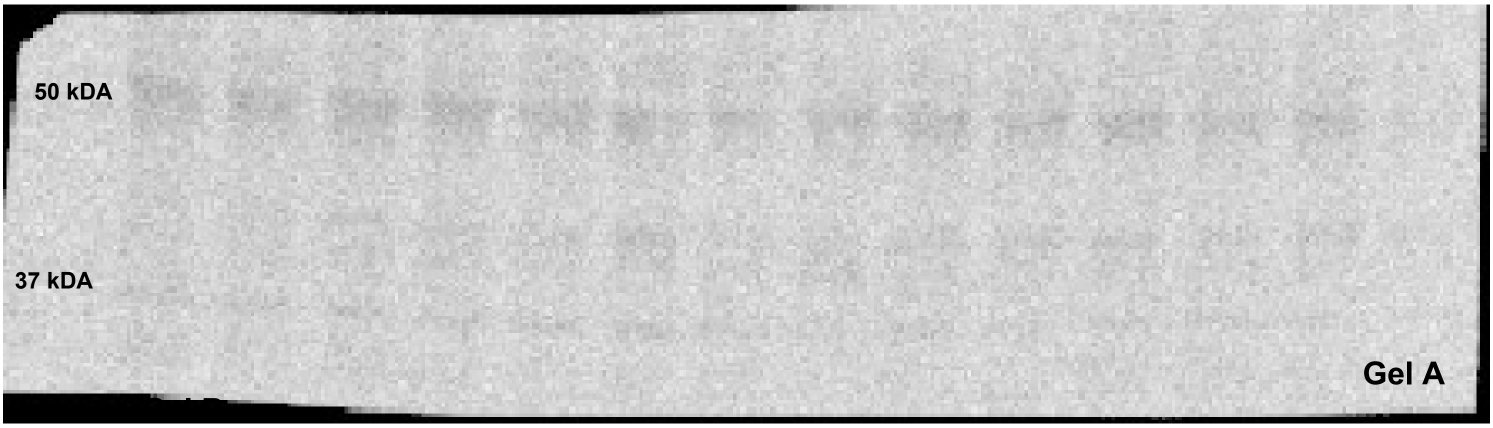

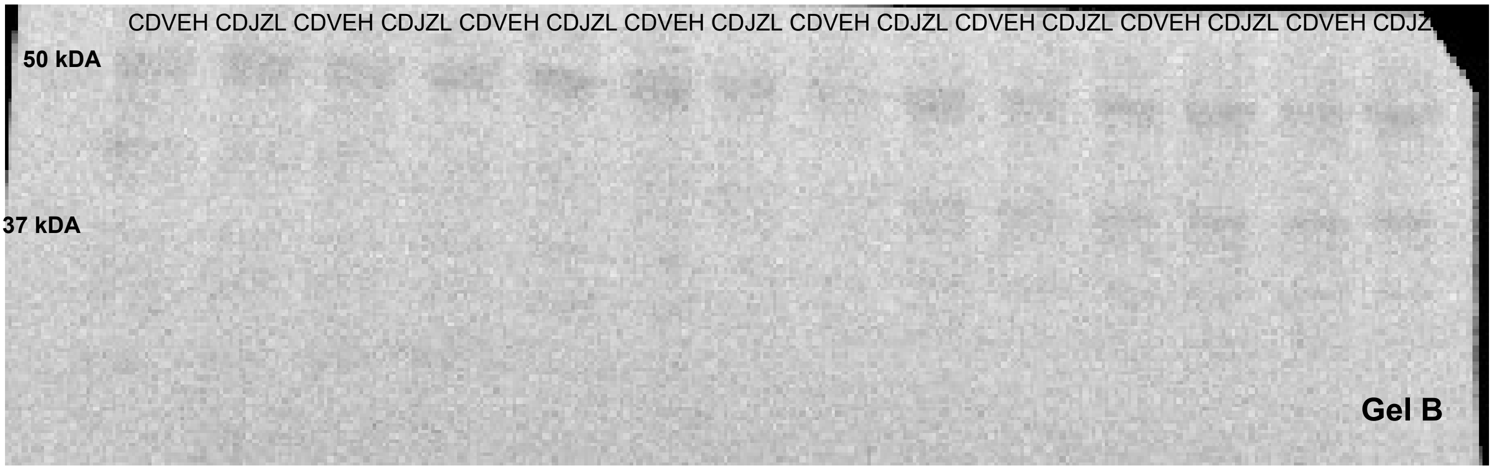

Supplement: Figure 4—figure supplement 1—source data 2. [file elife-72560-fig4-figsupp1-data2.pdf]
