# Supplementary material for: Cannabinoid signaling modulation through JZL184 restores key phenotypes of a mouse model for Williams–Beuren syndrome
Source: eLife. 2022 Oct 11;11:e72560. doi: 10.7554/eLife.72560 (PMC9553213; doi:10.7554/eLife.72560)
Supplement: Supplementary file 1. — Statistical significance was calculated by Student’s t-test. *p < 0.05; **p < 0.01 (genotype effect). Data are expressed as mean ± standard error of the mean (SEM). [file elife-72560-supp1.docx]

**Supplementary File 1**

|  | **WT** | **CD** |
| --- | --- | --- |
| ***Telencephalon*** |  |  |
| **Amygdala** |  |  |
| Anterior | 336± 21 | 368 ± 32 |
| Basolateral | 427 ± 20 | 495 ± 24* |
| Central | 464 ± 17 | 600 ± 54* |
| Medial | 196 ± 18 | 233 ± 10 |
| Cortical amygdaloid nu | 552 ± 24 | 506 ± 43 |
| **Cortex** |  |  |
| Auditory | 243 ± 17 | 272 ± 13 |
| Cingular | 453 ± 36 | 479 ± 23 |
| Frontal | 578 ± 37 | 552 ± 36 |
| Ectorhinal | 414 ± 26 | 449 ± 32 |
| Entorhinal | 378 ± 20 | 419 ± 29 |
| Motor | 375 ± 29 | 411 ± 22 |
| Perirhinal | 384 ± 24 | 422 ± 24 |
| Piriform | 288 ± 13 | 334 ± 27 |
| Somatosensory | 298 ± 25 | 325 ± 21 |
| Visual | 298 ± 18 | 324 ± 22 |
| **Hippocampus** |  |  |
| CA1 |  |  |
| Oriens | 506 ± 31 | 531 ± 51 |
| Pyramidal | 443 ± 23 | 403 ± 38 |
| Radiatum | 674 ± 42 | 614 ± 54 |
| CA3 |  |  |
| Oriens | 743 ± 43 | 731 ± 59 |
| Pyramidal | 424 ± 37 | 350 ± 25 |
| Radiatum | 823 ± 47 | 796 ± 59 |
| Dentate Gyrus |  |  |
| Molecular | 610 ± 31 | 536 ± 50 |
| Polymorphic | 594 ± 28 | 470 ± 35* |
| Granular | 246 ± 25 | 176 ± 13* |
| Ventral subiculum | 1000 ± 42 | 986 ± 73 |
| **Basal ganglia** |  |  |
| Globus pallidus | 1958 ± 140 | 2061 ± 205 |
| Striatum | 577 ± 48 | 606 ± 44 |
| ***Diencephalon*** |  |  |
| Basal nucleus | 351 ± 25 | 426 ± 62 |
| Medial septum | 477 ± 23 | 488 ± 17 |
| ***Rhinencephalon*** |  |  |
| Olfactory bulb (glomerular) | 423 ± 17 | 460 ± 27 |
| ***Rhomboencephalon*** |  |  |
| Dorsal raphe | 310 ± 29 | 361 ± 30 |
| ***Mesencephalon*** |  |  |
| Periaqueductal Gray | 417 ± 39 | 477 ± 27 |
| Substantia nigra | 2248 ± 115 | 2350 ± 136 |
| ***Metencephalon*** |  |  |
| **Cerebellum** |  |  |
| Cerebelar gray matter | 1447 ± 80 | 1392 ± 92 |
