# Supplementary material for: Cannabinoid signaling modulation through JZL184 restores key phenotypes of a mouse model for Williams–Beuren syndrome
Source: eLife. 2022 Oct 11;11:e72560. doi: 10.7554/eLife.72560 (PMC9553213; doi:10.7554/eLife.72560)
Supplement: Supplementary file 2. — Statistical significance was calculated by Student’s t-test. *p < 0.05; **p < 0.01 (genotype effect). Data are expressed as mean ± standard error of the mean (SEM). [file elife-72560-supp2.docx]

**Supplementary File 2**

|  | **WT** | **CD** |
| --- | --- | --- |
| *Telencephalon* |  |  |
| **Amygdala** |  |  |
| Anterior | 147 ± 18 | 163 ± 12 |
| Basolateral | 226 ± 23 | 322 ± 22 ** |
| Central | 95 ± 15 | 157 ± 24 * |
| Medial | 54 ± 8 | 87 ± 18 |
| Cortical amygdaloid nu | 188 ± 20 | 213 ± 19 |
| **Cortex** |  |  |
| Auditory | 112 ± 14 | 140 ± 12 |
| Cingular | 191 ± 17 | 224 ± 20 |
| Frontal | 216 ± 20 | 252 ± 30 |
| Ectorhinal | 219 ± 12 | 249 ± 22 |
| Entorhinal | 236 ± 17 | 281 ± 21 |
| Motor | 170 ± 12 | 190 ± 17 |
| Perirhinal | 232 ± 16 | 303 ± 31 |
| Piriform | 198 ± 19 | 257 ± 13 * |
| Somatosensory | 117 ± 10 | 141 ± 11 |
| Visual | 121 ± 13 | 173 ± 17 * |
| **Hippocampus** |  |  |
| CA1 |  |  |
| Oriens | 157 ± 13 | 226 ± 26 * |
| Pyramidal | 213 ± 18 | 263 ± 50 |
| Radiatum | 171 ± 12 | 232 ± 23* |
| CA3 |  |  |
| Oriens | 181 ± 23 | 199 ± 23 |
| Pyramidal | 240 ± 29 | 255 ± 25 |
| Radiatum | 237 ± 30 | 247 ± 38 |
| Dentate Gyrus |  |  |
| Molecular | 144 ± 23 | 127 ± 11 |
| Polymorphic | 224 ± 16 | 213 ± 14 |
| Granular | 243 ± 51 | 250 ± 31 |
| Ventral subiculum | 208 ± 20 | 204 ± 17 |
| **Basal ganglia** |  |  |
| Globus pallidus | 1533 ± 64 | 1627 ± 82 |
| Striatum | 203 ± 14 | 222 ± 16 |
| *Diencephalon* |  |  |
| Basal nucleus | 112 ± 9 | 147 ± 16 |
| Medial septum | 158 ± 11 | 161 ± 16 |
| *Rhinencephalon* |  |  |
| Olfactory bulb (glomerular) | 605 ± 34 | 757 ± 18 ** |
| *Rhomboencephalon* |  |  |
| Dorsal raphe | 64 ± 10 | 67 ± 7 |
| *Mesencephalon* |  |  |
| Periaqueductal Gray | 71 ± 9 | 101 ± 16 |
| Substantia nigra | 1760 ± 114 | 1694 ± 82 |
| *Metencephalon* |  |  |
| **Cerebellum** |  |  |
| Cerebelar gray matter | 380 ± 34 | 372 ± 37 |
