# Supplementary material for: Cannabinoid signaling modulation through JZL184 restores key phenotypes of a mouse model for Williams–Beuren syndrome
Source: eLife. 2022 Oct 11;11:e72560. doi: 10.7554/eLife.72560 (PMC9553213; doi:10.7554/eLife.72560)
Supplement: Supplementary file 3. — Interventricular septum diastolic (IVSd), left ventricular posterior wall thickness diastolic (LVPWd), LV end-diastolic diameter (LVDd), and left ventricular mass diastolic (Lvmass) measurement relatives to body weight (BW) (WT VEH, n = 8; WT JZL184, n = 7; CD VEH, n = 7; CD JZL184, n = 7). Statistical significance was calculated by Newman–Keuls post hoc test following two-way analysis of variance (ANOVA) ***p < 0.001 (genotype effect). Data are expressed as mean ± standard error of the mean (SEM). [file elife-72560-supp3.docx]

**Supplementary File 3**

|  | **Echocardiogram** | | | |
| --- | --- | --- | --- | --- |
|  | WT VEH | WT JZL 184 | CD VEH | CD JZL 184 |
| IVSd/BW | 0.0181 ± 0.0002 | 0.0184 ± 0.0009 | 0.0259 ± 0.0010 *** | 0.0237 ± 0.0010 |
| LVPWd/BW | 0.0178 ± 0.0001 | 0.0186 ± 0.0008 | 0.0244 ± 0.0007 *** | 0.0227 ± 0.0012 |
| LVDd/BW | 0.1232 ± 0.0060 | 0.1349 ± 0.0065 | 0.1499 ± 0.0078 | 0.1378 ± 0.0088 |
| Lvmass/BW | 0.0224 ± 0.0005 | 0.0233 ± 0.0011 | 0.0281 ± 0.0006 *** | 0.0263 ± 0.0009 |
