# Supplementary material for: Cannabinoid signaling modulation through JZL184 restores key phenotypes of a mouse model for Williams–Beuren syndrome
Source: eLife. 2022 Oct 11;11:e72560. doi: 10.7554/eLife.72560 (PMC9553213; doi:10.7554/eLife.72560)
Supplement: Supplementary file 4. — Pathways were obtained from Reactome database and expression changes are expressed as log2 ratios of fold-changes between CD mice treated for 10 days with JZL184 (8 mg/kg) and CD or WT littermates treated with vehicle. [file elife-72560-supp4.docx]

**Supplementary File 4**

|  | **Genes** | **WT VEH vs CD VEH**  **(log_2_FC)** | **CD JZL184 vs CD VEH**  **(log_2_FC)** |
| --- | --- | --- | --- |
| ***Inflammasomes*** | | | |
|  | | | |
|  | *App* | 0,2417 | -- |
|  | *Bcl2* | -0,3603 | -- |
|  | *Casp1* | 0,7794 | -0,6407 |
|  | *Hmox1* | 1,7239 | -1,6503 |
|  | *Hsp90ab1* | -0,3290 | -- |
| ***Striated muscle contraction*** | | | |
|  |  |  |  |
|  | *Acta1* | -2,2394 | -- |
|  | *Actc1* | -0,3203 | -- |
|  | *Actn2* | -0,4700 | 0,4274 |
|  | *Des* | -0,2817 | -- |
|  | *Dmd* | -0,7061 | 0,4919 |
|  | *Myh6* | -0,4482 | 0,4619 |
|  | *Myl1* | -0,4362 | -- |
|  | *Neb* | -1,3141 | -- |
|  | *Tmod1* | -0,3742 | 0,3058 |
|  | *Tmod3* | 0,3594 | -0,3873 |
|  | *Tmod4* | -0,7021 | -- |
|  | *Tpm3* | 0,4519 | -0,4196 |
|  | *Tpm4* | 0,5041 | -0,6803 |
|  | *Ttn* | -1,0692 | -- |
|  | *Vim* | 0,3686 | -0,3853 |
| ***Smooth muscle contraction*** | | | |
|  |  |  |  |
|  | *Anxa1* | 0,6990 | -0,7698 |
|  | *Anxa2* | 0,4466 | -0,5849 |
|  | *Cald1* | 0,2627 | -0,3666 |
|  | *Gucy1a1* | 0,4715 | -0,3198 |
|  | *Gucy1b1* | 0,5693 | -0,5076 |
|  | *Myh11* | 0,6968 | -0,6403 |
|  | *Myl12b* | 0,4941 | -0,4893 |
|  | *Myl6* | 0,6275 | -0,7171 |
|  | *Myl9* | 0,8271 | -0,8810 |
|  | *Mylk* | 0,3030 | -0,3802 |
|  | *Sorbs1* | -0,6050 | 0,6412 |
|  | *Tpm3* | 0,4519 | -0,4196 |
|  | *Tpm4* | 0,5041 | -0,6803 |
|  | *Trim72* | -0,4174 | 0,3966 |
|  | *Vcl* | -0,4783 | 0,3867 |
| ***Cardiac conduction*** | | | |
|  |  |  |  |
|  | *Abcc9* | -0,5537 | 0,3503 |
|  | *Akap9* | -0,3981 | 0,3024 |
|  | *Atp1a1* | -0,4184 | 0,2859 |
|  | *Atp1b1* | -0,3288 | 0,2672 |
|  | *Atp1b3* | 0,2214 | -0,2271 |
|  | *Atp2a2* | -0,3754 | 0,5094 |
|  | *Atp2b4* | 0,3403 | -0,2917 |
|  | *Cacna1c* | -0,6323 | 0,6503 |
|  | *Cacna2d2* | -0,9143 | 0,8180 |
|  | *Cacnb2* | -0,6545 | 0,3922 |
|  | *Camk2a* | -0,2960 | 0,3635 |
|  | *Camk2b* | -0,6566 | 0,2853 |
|  | *Camk2d* | -0,3975 | 0,3553 |
|  | *Camk2g* | -0,3698 | 0,3931 |
|  | *Corin* | -0,3644 | 0,3988 |
|  | *Fgf13* | -0,3787 | 0,4486 |
|  | *Fxyd1* | 0,1851 | -0,1869 |
|  | *Fxyd6* | 0,6705 | -- |
|  | *Gata4* | -0,3241 | 0,3434 |
|  | *Hipk1* | -0,1958 | -- |
|  | *Hipk2* | -0,5799 | 0,6617 |
|  | *Kcnd3* | -0,5831 | 0,6359 |
|  | *Kcnip2* | -0,2481 | 0,2084 |
|  | *Kcnj11* | -0,2249 | 0,3827 |
|  | *Kcnj12* | -0,3615 | 0,3972 |
|  | *Kcnk13* | 0,9408 | -- |
|  | *Kcnk3* | -0,2508 | 0,3611 |
|  | *Kcnk6* | 0,2941 | -0,3586 |
|  | *Kcnq1* | -0,3117 | 0,4610 |
|  | *Prkaca* | -0,3252 | 0,3564 |
|  | *Rangrf* | 0,3055 | -0,2900 |
|  | *Ryr2* | -0,4468 | 0,6442 |
|  | *Scn10a* | -0,7584 | 0,7339 |
|  | *Scn1b* | -0,5079 | -- |
|  | *Scn5a* | -0,8295 | 0,5890 |
|  | *Scn7a* | 0,2430 | -0,2567 |
|  | *Slc8a1* | -0,6317 | 0,7132 |
|  | *Slc8a2* | -0,9611 | -- |
|  | *Sri* | 0,3665 | -0,4116 |
| ***G protein mediated events*** | | | |
|  |  |  |  |
|  | *Adcy1* | -1,3572 | -- |
|  | *Adcy5* | -0,5638 | 0,3931 |
|  | *Adcy6* | -0,4475 | 0,4569 |
|  | *Adcy9* | -0,4346 | 0,5669 |
|  | *Camk2a* | -0,2960 | 0,3635 |
|  | *Camk2b* | -0,6566 | 0,2853 |
|  | *Camk2d* | -0,3975 | 0,3553 |
|  | *Camk2g* | -0,3698 | 0,3931 |
|  | *Gnai1* | 0,6013 | -- |
|  | *Gnai2* | 0,3204 | -- |
|  | *Gnai3* | 0,2786 | -0,4056 |
|  | *Mapk1* | -0,2055 | 0,2038 |
|  | *Nbea* | -0,3583 | 0,4002 |
|  | *Pde1a* | 0,4527 | -0,4021 |
|  | *Pde1b* | 0,5506 | -0,4399 |
|  | *Pde1c* | -0,6176 | 0,4260 |
|  | *Plcb1* | 0,5096 | -0,5229 |
|  | *Prkaca* | -0,3252 | 0,3564 |
|  | *Prkacb* | -0,1977 | -- |
|  | *Prkar1a* | -0,3322 | -- |
|  | *Prkar2a* | -0,3848 | 0,3006 |
|  | *Prkca* | -0,2374 | 0,3290 |
| ***G protein activation*** | | | |
|  |  |  |  |
|  | *Gnb1* | 0,2388 | -0,1769 |
|  | *Gnb2* | 0,2207 | -- |
|  | *Gnb4* | 0,2689 | -0,2160 |
|  | *Gng11* | 0,6453 | -0,7743 |
|  | *Gng12* | 0,2293 | -- |
|  | *Gngt2* | 0,6693 | -0,7298 |
| ***Activation of AMPK downstream of NMDARS*** | | | |
|  |  |  |  |
|  | *Prkab2* | -0,7461 | 0,5766 |
|  | *Prkaa2* | -0,6268 | 0,6166 |
|  | *Mapt* | -0,4443 | 0,3965 |
|  | *Prkag2* | -0,3349 | 0,2501 |
|  | *Prkag3* | -0,7713 | -- |
|  | *Tuba1a* | 0,5145 | -0,5953 |
|  | *Tubb2a* | 0,3829 | -0,5595 |
|  | *Tuba1b* | 0,3469 | -0,3411 |
|  | *Prkab1* | 0,3043 | -- |
| ***mTOR signalling*** | | | |
|  |  |  |  |
|  | *Cab39* | -0,2698 | -- |
|  | *Cab39l* | 0,2145 | -- |
|  | *Eef2k* | -0,3008 | 0,2888 |
|  | *Eif4ebp1* | 0,2307 | -- |
|  | *Eif4g1* | -0,4307 | 0,4191 |
|  | *Lamtor2* | 0,2009 | -0,1774 |
|  | *Lamtor4* | 0,3481 | -0,3173 |
|  | *Lamtor5* | 0,3299 | -0,3298 |
|  | *Mtor* | -0,3446 | 0,4111 |
|  | *Prkaa2* | -0,6268 | 0,6166 |
|  | *Prkab1* | 0,3043 | -- |
|  | *Prkab2* | -0,7461 | 0,5766 |
|  | *Prkag2* | -0,3349 | 0,2501 |
|  | *Prkag3* | -0,7713 | -- |
|  | *Rps6* | 0,3709 | -0,4002 |
|  | *Rptor* | -0,3106 | 0,3728 |
|  | *Rragc* | -0,2263 | -- |
|  | *Rragd* | -0,5970 | 0,3825 |
|  | *Tsc1* | -0,3522 | 0,3877 |
|  | *Tsc2* | -0,2759 | 0,3345 |
